# Supplementary material for: Preferences of people living with HIV for differentiated care models in Kenya: A discrete choice experiment
Source: PLoS One. 2021 Aug 25;16(8):e0255650. doi: 10.1371/journal.pone.0255650 (PMC8386850; doi:10.1371/journal.pone.0255650)
Supplement: Attachment — Submitted filename: 20-24745-Review comments.docx [file pone.0255650.s002.docx]

I would like to acknowledge that I have reviewed your paper titled “Preferences of People Living with HIV for Differentiated Care Models in Kenya: A Discrete Choice Experiment.” It is a very important topic as countries aim for patient-centered care as well as adherence to ART among PLHIV.

Below are a few comments that the authors can consider.

1. Although the attribute identification for the DCE is supported by evidence (Literature review and qualitative interviews), the authors do not justify how the final seven attributes and their levels were arrived at. For instance, its not clear how many attributes were identified from the literature review, how many (more or less) attributes were identified from the qualitative interviews and what process was involved in perhaps reducing the number of attributes or having a consensus about the attribute levels. These are important aspects of reporting conjoint analyses (Bridges et al., 2011). Please clarify
2. It is not clear to me why the example choice task, Table 2, has four attributes instead of the seven. Does this mean the “twelve different, randomly generated” questionnaire versions had a different number/set of attributes? (If I interpret this correctly, 12 blocks were generated each with 10 choice tasks – If this is the case then each task, irrespective of the block would have all the seven attributes and three alternatives). On the other hand, the authors might have opted for partial profiles, however, this has not been described anywhere. Please explain.
3. Given that the study took place in one facility, it is surprising that the study questionnaire was not piloted. How can we guarantee that respondents could easily understand the attributes and levels, the number of attributes and alternatives in each task and generally the number of choice tasks per questionnaire did not result in respondent cognitive burden? Please clarify.
4. Table 4, I suggest the units (years, KSH/month and minutes) be bracketed to avoid confusion. For example, the travel time unit could be interpreted as presenting the minimum value (which can be abbreviated as min).
5. The authors state that they did not meet their original sample size goal. This is not true. They actually surpassed their target minimum sample size of 67 respondents, however, this sample size was only optimized to examine main effects but not all possible two-way interactions. To have enabled an examination of these, ‘c’ should have been equal to the largest product of levels of any two attributes (in this case 12). Rephrase this limitation.
