# Supplementary material for: Preferences of people living with HIV for differentiated care models in Kenya: A discrete choice experiment
Source: PLoS One. 2021 Aug 25;16(8):e0255650. doi: 10.1371/journal.pone.0255650 (PMC8386850; doi:10.1371/journal.pone.0255650)
Supplement: Attachment — Submitted filename: Response to reviewers.docx [file pone.0255650.s003.docx]

Sagar Dommaraju

University of California-San Francisco 1600 Divisadero St

San Francisco, CA, USA 94115 C| 630-956-1646

E| drajus.88@gmail.com

Dr. Joerg Heber

Editor-in-Chief

PLOS One

March 13, 2021

Dear Dr. Heber and the reviewers,

Thank you for considering our manuscript for publication in your journal. Each comment from both reviewers were very helpful, especially in clarifying the methodology for our study. We addressed each comment and incorporated changes to our manuscript. Please find our responses below. Thank you.

Reviewer 1:

1. Although the attribute identification for the DCE is supported by evidence (Literature review and qualitative interviews), the authors do not justify how the final seven attributes and their levels were arrived at. For instance, its not clear how many attributes were identified from the literature review, how many (more or less) attributes were identified from the qualitative interviews and what process was involved in perhaps reducing the number of attributes or having a consensus about the attribute levels. These are important aspects of reporting conjoint analyses (Bridges et al., 2011). Please clarify.

All seven attributes in our study were identified from the literature. These attributes were confirmed as key components of a differentiated service delivery model with informants (researchers, healthcare workers) in Kenya who work with the HIV+ population. Of the original seven attributes that were identified, all were included in the final study. We did not identify any additional attributes that were later excluded. This explanation has been added to the text to clarify the process.

2. It is not clear to me why the example choice task, Table 2, has four attributes instead of the seven. Does this mean the “twelve different, randomly generated” questionnaire versions had a different number/set of attributes? (If I interpret this correctly, 12 blocks were generated each with 10 choice tasks – If this is the case then each task, irrespective of the block would have all the seven attributes and three alternatives). On the other hand, the authors might have opted for partial profiles, however, this has not been described anywhere. Please explain.

Thank you for this comment. We opted for a partial profile design based on findings from Chrzan et al wherein respondents had difficulty cognitively processing choice tasks with more than six attributes. We have added an explanation for this design decision in the methods section.

3. Given that the study took place in one facility, it is surprising that the study questionnaire was not piloted. How can we guarantee that respondents could easily understand the attributes and levels, the number of attributes and alternatives in each task and generally the number of choice tasks per questionnaire did not result in respondent cognitive burden? Please clarify.

We addressed this very real concern by having the researcher present each choice task verbally and then allowing the patient to read it. This was done in a standardized way. For example, a researcher might present a choice task as follows: “If these care models were your only options, which one would you choose? The first option is a clinic with ART refills every 3 months, viral load samples at a health facility, and an individual support system. The second option is…” We have added an explanation for this process in the methods section.

4. Table 4, I suggest the units (years, KSH/month and minutes) be bracketed to avoid confusion. For example, the travel time unit could be interpreted as presenting the minimum value (which can be abbreviated as min).

I’ve added brackets to the units in Table 4 to improve clarity, as you suggested.

5. The authors state that they did not meet their original sample size goal. This is not true. They actually surpassed their target minimum sample size of 67 respondents, however, this sample size was only optimized to examine main effects but not all possible two-way interactions. To have enabled an examination of these, ‘c’ should have been equal to the largest product of levels of any two attributes (in this case 12). Rephrase this limitation.

This is a great point. I have changed the wording in the methods section to reflect that the n=67 sample size calculation is for main effects. I also rephrased the limitation at the end of our discussion section as you suggested.

Reviewer 2:

1. In the introduction section, provide a brief context of the Kenyan health system and how the current HIV care is delivered. This will help explain why DSD models in Kenya are important. Just a paragraph would do to help any readers to understand the context, understand why a DCE was needed, and understand the policy context. State why the DCE was better than any other method out there.

I added a paragraph to the introduction section describing how HIV care has been managed over the past decade in Kenya. Later in the introduction, I also added a few sentences to explain why a DCE is the preferred method for this study.

2. The methodology section is not clear. It omits a lot of crucial information that helps the reader understand what was done.

In addressing the comments below, we hope to have clarified our methodology and provided important context on how the study was designed and how the data was analyzed.

3. “Patients were introduced to the study during their clinician visit if they met criteria as a stable patient. One of three researchers approached each interested patient to obtain oral consent, and administered the questionnaire containing sociodemographic information and the ten choice tasks in the language of the patient’s choosing. Patient IDs were also collected to obtain additional data from the EMR. Detailed field notes and observations were taken in tandem. Basic demographic information was collected from all participants: age, gender, education level, average monthly income, and average travel time to the clinic.” – This piece of text should be put in the right place which describes data collection. This paragraph should come after sample size calculation because you talk about 10 choice tasks being administered while you haven’t described attributes, levels, experimental design, questionnaire format, and sampling. Therefore, it should be placed further down the methodology section preferably after sampling/sample size calculation.

This paragraph was moved as you suggested.

4. I can see that from Table 2, the DCE was unlabelled with three hypothetical alternatives without an opt-out. You need to state this in the text before the sentence “Questionnaires were produced by using the Choice-based Conjoint feature of Sawtooth Software™. Justify in the text why you opted for a forced-choice format i.e. why was the opt-out not needed.

It has now been clearly stated that our DCE uses a forced-choice format. Rationale for doing so has been included in the paragraph that you indicated.

5. In the construction of choice tasks, did you use partial profiles? Because Table 1 shows 7 attributes while Table 2 shows only 4 attributes. If so, state this in the text why partial profiles were used instead of full profiles.

Thank you for this comment. We opted for a partial profile design based on findings from Chrzan et al wherein respondents had difficulty cognitively processing choice tasks with more than six attributes. We have added an explanation for this design decision in the methods section.

6. Why was an orthogonal design appropriate for this study while there are far better designs such as efficient and Bayesian efficient designs? State this in the text. Also clearly state that the orthogonal design was a main effects model.

We used sawtooth software to generate the choice experiment design. Sawtooth generates the design by sampling from a subset of the full-choice designs for each respondent while ensuring level balance and near-orthogonality within each respondent’s profile, this allows for the generation of up to 999 blocks, and using a unique randomized design for each respondent reduces context effects (Reference Reed Johnson. Constructing Experimental Designs for Discrete-Choice Experiments: Report of the ISPOR Conjoint Analysis Experimental Design Good Research Practices Task Force). Sawtooth software is widely used for DCE design, administration and analysis, as a result we chose this approach for our experiment design. We have added these details to the manuscript.

7. The experimental design generated 10 choice tasks which were put into the questionnaire.

This phrasing has been added to the text.

8. Were the attributes and levels clearly explained to participants? State this in the text

We addressed this concern by having the researcher present each choice task verbally and then allowing the patient to read it. This was done in a standardized way. For example, a researcher might present a choice task as follows: “If these care models were your only options, which one would you choose? The first option is a clinic with ART refills every 3 months, viral load samples at a health facility, and an individual support system. The second option is…” We have added an explanation for this process in the methods section.

9. “used parametric and non-parametric tests to summarize all sociodemographic information using R version 3.2.3 [19]”. State these tests?

We listed the exact tests used to summarize the sociodemographic variables.

10. “importances” Relative importance would be a better?

The wording has been changed as you suggested.

11. “In addition, average importances are calculated and represent the relative importance of each attribute within the experiment. Average importances are presented as percentages and can be interpreted as how important each attribute is for a patient when making a decision regarding their preferred DSD model [17].” Clearly state in the text in the methodology section the method was used to calculate relative importance estimates.

Per Orme et al, in standard probability analysis the average importance of an attribute is calculated by dividing its utility range (i.e. the utility of the highest level minus the utility of the lowest level) by the sum total lof utility ranges of all attributes. These calculations are done automatically by Sawtooth Software. This explanation has been added to the text.

12. Write out the utility functions so we can see the model structure and coding of the attributes. What coding was used? Dummy coding or effects coding? Mention this in the text. It’s not clear at all.

The HB model in Sawtooth has two levels: At the upper level it is assumed that individuals’ vectors of part-worths are drawn from a multivariate normal distribution. At the lower level, a logit model is assumed for each individual, where the utility of each alternative is the sum of the part-worths of its attribute levels, and the respondent’s probability of choosing each alternative is equal to its utility divided by the sum of utilities for the alternatives in that choice set. Several Markov chain Monte Carlo (MCMC) simulations of an algorithm using these model estimates generates the part-worths for the individual, the mean for the population and variances and covariances.

We have now included these details in the manuscript and added a reference to sawtooth HB analyses methods and formulae for readers to review.

13. What distributional assumptions did you make in your Hierarchical Bayes model for each attribute? Normal, lognormal, uniform? State this in text

This is addressed above in details regarding sawtooth analysis methods.

14. What did the constant (alternative specific constant (asc)) represent in your utility function since your DCE adopted a forced choice format?

This is addressed above in details regarding sawtooth analysis methods.

15. Tables 5 and 7: Could you clearly indicate which were the base levels (omitted categories/reference category) of the attributes? I can see you have used effects coding if I assume the base levels are first levels that appear for each attribute as the coefficient of the base level will be the negative sum of the included-category coefficients. However, you have not stated anywhere in the manuscript whether effects coding was used. You have to state this to make it easier for the reader.

We have now stated in the methods that effects coding was used.

16. In Tables 5 and 7, provide the confidence intervals or standard errors for both the coefficients (relative utility) and standard deviations. There is only one confidence interval. Furthermore, what is the coefficient and standard deviation of your alternative specific constant (asc) and what does its coefficient represent?

Ninety-five percent confidence intervals have been added to Tables 5 and 7 for the standard deviations. The alternative specific constant has been addressed above in details regarding sawtooth analysis methods.

17. Table 6. Which method was used to calculate the relative importance estimates (what you call “average importances”) probability analysis? Or Partial log likehood analysis? State this in the methods section and what it means.

Average importances are calculated as indicated above, using estimated from HB analysis (Reference: Sawtooth software Interpreting Conjoint Analysis Data series: Interpreting Conjoint Analysis Data)

18. The wording of the manuscript needs to be improved to reflect the fact that you are reporting a DCE.

In addressing the comments above, we hope to have clarified our methodology and to now better reflect that we are reporting a DCE.

19. Also place your study results in the context of Universal health coverage reforms in Kenya.

We have added a paragraph in the discussion section to highlight how the fast-track, multi-month scripting model preferred by patients in our study may also help alleviate system inefficiencies, which would be beneficial for Kenya’s goal of providing universal healthcare to patients.

Sincerely,

Sagar Dommaraju, MSc

Student, Department of Global Health University of California-San Francisco
