# Supplementary material for: Preferences of people living with HIV for differentiated care models in Kenya: A discrete choice experiment
Source: PLoS One. 2021 Aug 25;16(8):e0255650. doi: 10.1371/journal.pone.0255650 (PMC8386850; doi:10.1371/journal.pone.0255650)
Supplement: Attachment — Submitted filename: Response to reviewers.docx [file pone.0255650.s004.docx]

Sagar Dommaraju

University of California-San Francisco 1600 Divisadero St

San Francisco, CA, USA 94115

C| 630-956-1646

E| drajus.88@gmail.com

Dr. Joerg Heber

Editor-in-Chief

PLOS One

April 28, 2021

Dear Dr. Heber and the reviewers,

Thank you for considering our manuscript for publication in your journal. Each comment from the reviewers in the second round of editing was helpful, especially in clarifying the methodology for our study. We addressed each comment and incorporated changes to our manuscript. Please find our responses below. Thank you.

Reviewer 1: (No response).

Reviewer 2:

1. I am happy with the corrections. I will just request the authors to quickly write down the utility functions and insert it in the main text, after the paragraph explaining the HB model. Then submit the manuscript to the editor for publishing.

In addition to describing in words the functions for relative utility and attribute importance, we added the utility function used in our model which was derived from a textbook chapter on conjoint analysis by Rao et al. This is the utility function used by Sawtooth to calculate the relative utilities in our study. We hope that adding this function clarifies our approach and completes the revision of our methodology.

The additional reference mentioned above has been added to our reference list.

Sincerely,

Sagar Dommaraju, MSc

Student, Department of Global Health University of California-San Francisco
