# Supplementary material for: Preferences of people living with HIV for differentiated care models in Kenya: A discrete choice experiment
Source: PLoS One. 2021 Aug 25;16(8):e0255650. doi: 10.1371/journal.pone.0255650 (PMC8386850; doi:10.1371/journal.pone.0255650)
Supplement: Attachment — Submitted filename: Response to reviewers.docx [file pone.0255650.s006.docx]

Sagar Dommaraju

University of California-San Francisco 1600 Divisadero St

San Francisco, CA, USA 94115

C| 630-956-1646

E| drajus.88@gmail.com

Dr. Joerg Heber

Editor-in-Chief

PLOS One

July 20, 2021

Dear Dr. Heber and the reviewers,

Thank you for considering our manuscript for publication in your journal. Each comment from the editor in the third round of editing was helpful, especially in clarifying the methodology for our study. We addressed each comment and incorporated changes to our manuscript. Please find our responses below. Thank you.

Reviewer 1:

1. The novelty of the research paper is very excellent; selection of the variables and attributes is patrsimonial state model. As per the literature, any model would be constructed or formulated by the author; the model should be expressive form and define the state variables of our objective of interest. I have carefully examined your model, you are unable to define the state variables in the model structure .Although, your formulated model will not be substantiate the state variables because not enough to propagate the state variable attributes for estimation of likelihoods based on numerical simulation. During the process of review, the following mathematical eqn affixed for your information and requested to include in your research paper

𝑈(𝑥𝑗𝑡) = 𝐷𝑡1𝑈𝑡1 + 𝐷𝑡2𝑈𝑡2 + ⋯ + 𝐷𝑡𝑟𝑡𝑈𝑡	(1.1)

Ut_((xjt) ~)	N (〖Dt〗_i 〖Ut〗_i )

The effect of each attributed of DCE was modeled by

Ut_xjt=Di((t1+t2..tn)/k)*Ui	(1.2)

Where Di= The discrete value assigned for each of ith attributes

‘K’ is the discrete level of Xt

t1+t2..tn is the sum of the attributes at nth level

The above equations defining our state variables have been added to our Methods section as requested.

2. Table 7 represented the Normalized average utilities of all levels by gender. A higher magnitude indicates a stronger preference, while a positive or negative value .plz estimate the likelihood on each attributes based on the DCE state variables

It is unclear what the reviewer is suggesting in this comment. If by “likelihood on each attribute” he is referring to the importance of each attribute by gender, there were no significant differences between genders when it comes to weight given to each attribute when choosing between care models. That is, the importance of each attribute for men and women were not significantly different from the importances of the cohort at large (listed in Table 6). As this was not relevant to our discussion, it was not included in our results section. I hope this clarifies the issue.

3. Table 3 Please correlate the selected attributes from weighting time for receiving ART drugs ( weighting time is the state variable)

Table 3 represents the sociodemographic information and baseline characteristics of our study population; there is no variable called “weighting time” in this table. I believe you are referring to Table 5, and specifically the attribute “frequency of ART refills.” If so, the weight of all attributes—and thus the corresponding relative utilities assigned to each level—are relative to the attribute with the highest importance. In our study, the most important attribute for patients when deciding between care models would be “location of clinical review,” not “frequency of ART refills.” I hope this clarifies your question.

4. The optimization of your model is reached maximum epoch, but interaction effect of the attributes is not mentioned in any ware either in result and discussion part.

When describing limitations of our study in our discussion section, we specifically mention that our study was not powered to examine two-way interactions between attributes. I have added an additional sentence to this paragraph to clarify, and I hope this addresses your comment.

Sincerely,

Sagar Dommaraju, MSc

Student, Department of Global Health University of California-San Francisco
