# Supplementary material for: Preferences of people living with HIV for differentiated care models in Kenya: A discrete choice experiment
Source: PLoS One. 2021 Aug 25;16(8):e0255650. doi: 10.1371/journal.pone.0255650 (PMC8386850; doi:10.1371/journal.pone.0255650)
Supplement: Attachment — Submitted filename: reviewer comment.docx [file pone.0255650.s005.docx]

Dear author

I would like to acknowledge that I have reviewed your paper titled “Preferences of People Living with HIV for Differentiated Care Models in Kenya: A Discrete Choice Experiment.” It is a very important topic as countries aim for patient-centered care as well as health policy making decisions PLHIV (PONE-D-20-24745R2)

Below are a few comments that the authors can consider.

(i)The novelty of the research paper is very excellent; selection of the variables and attributes is patrsimonial state model. As per the literature, any model would be constructed or formulated by the author; the model should be expressive form and define the state variables of our objective of interest. I have carefully examined your model, you are unable to define the state variables in the model structure .Although, your formulated model will not be substantiate the state variables because not enough to propagate the state variable attributes for estimation of likelihoods based on numerical simulation. During the process of review, the following mathematical eqn affixed for your information and requested to include in your research paper

𝑈(𝑥𝑗𝑡) = 𝐷𝑡1𝑈𝑡1 + 𝐷𝑡2𝑈𝑡2 + ⋯ + 𝐷𝑡𝑟𝑡𝑈𝑡	(1.1)

$$\mathrm{Ut}_{\left( \mathrm{xjt} \right) \sim} N \left( {Dt}_{i} {Ut}_{i} \right)$$

The effect of each attributed of DCE was modeled by

$\mathrm{Ut}_{\mathrm{xjt}}=Di\left( \frac{t1+t2..tn}{k} \right)*Ui$	(1.2)

Where $Di=$ The discrete value assigned for each of i^th^ attributes

‘K’ is the discrete level of Xt

$t1+t2..tn$ is the sum of the attributes at n^th^ level

(ii) Table 7 represented the Normalized average utilities of all levels by gender. A higher magnitude indicates a stronger preference, while a positive or negative value .plz estimate the likelihood on each attributes based on the DCE state variables

iii) Table 3 Please correlate the selected attributes from weighting time for receiving ART drugs ( weighting time is the state variable)

The optimization of your model is reached maximum epoch, but interaction effect of the attributes is not mentioned in any ware either in result and discussion part.

Requested the Author,plz Rephrase the above limitation and comments .
