# Supplementary material for: Preferences of people living with HIV for differentiated care models in Kenya: A discrete choice experiment
Source: PLoS One. 2021 Aug 25;16(8):e0255650. doi: 10.1371/journal.pone.0255650 (PMC8386850; doi:10.1371/journal.pone.0255650)
Supplement: Attachment — Submitted filename: reviewer comments PLOS.docx [file pone.0255650.s007.docx]

Date: 2021-07-21 @19.23 GMT

Dear chief editor

Warm greetings ….!!!!

I have carefully reviewed the research paper bearing **No:PONE-D-20-24745R3: “Preferences of People Living with HIV for Differentiated Care Models in Kenya: A Discrete Choice Experiment”** Based on the comments made by the reviewers, the following key points noted for your reference and publication process.

(i) HIV is a scourge; an approximately 2.30 million population ended their lives in every year because due to poor resources, scarcity of antiretroviral therapy and economic burden of the drug manufacturing process .In this paradigm HIV research itself is debatable issue at International platform. Since way back in 1980 first case of HIV has recorded at worldwide, but till date we need more research for policy making, drug development and vaccination trail etc.
(ii) Eradication of HIV disease is a major challenge to us, many scientists eagerly conducted clinical, epidemiological and advanced research on novel HIV regimen. While on the micro and analytical research point of view we need some extensive research papers for citations and methodological process of new research work. However, HIV researcher and academician needs good research papers for their citation and inception of further research work conducted at globally. In spite of the advancement of HIV research , the present reviewed article is merit and meet our Journal requirements

As a academic editor, I strongly recommend this article for publication in our esteemed PLOS journal.

**Please consider the environment before you print this email.**

To Save our mother land..!!!

Best regards

Dr Basavarajaiah DM

Academic Editor-PLOS, KVAFSU(B),Bidar
